# Unique Molecular Identifier-Based High-Resolution HLA Typing and Transcript Quantitation Using Long-Read Sequencing

**DOI:** 10.3389/fgene.2022.901377

**Published:** 2022-06-13

**Authors:** Caleb Cornaby, Maureen C. Montgomery, Chang Liu, Eric T. Weimer

**Affiliations:** ^1^ Molecular Immunology Laboratory, McLendon Clinical Laboratories, UNC Health, Chapel Hill, NC, United States; ^2^ Department of Pathology and Immunology, Division of Laboratory and Genomic Medicine, School of Medicine, Washington University in St. Louis, St. Louis, MO, United States; ^3^ Department of Pathology and Laboratory Medicine, The University of North Carolina at Chapel Hill School of Medicine, Chapel Hill, NC, United States

**Keywords:** unique molecular identifier (UMI), nanopore, long-read, HLA, immunogenetics allele-specific expression, high-resolution HLA

## Abstract

HLA typing provides essential results for stem cell and solid organ transplants, as well as providing diagnostic benefits for various rheumatology, gastroenterology, neurology, and infectious diseases. It is becoming increasingly clear that understanding the expression of patient HLA transcripts can provide additional benefits for many of these same patient groups. Our study cohort was evaluated using a long-read RNA sequencing methodology to provide rapid HLA genotyping results and normalized HLA transcript expression. Our assay used NGSEngine to determine the HLA genotyping result and normalized mRNA transcript expression using Athlon2. The assay demonstrated an excellent concordance rate of 99.7%. Similar to previous studies, for the class I loci, patients demonstrated significantly lower expression of *HLA-C* than *HLA-A* and *-B* (Mann–Whitney U, *p* value = 0.0065 and *p* value = 0.0154, respectively). In general, the expression of class II transcripts was lower than that of class I transcripts. This study demonstrates a rapid high-resolution HLA typing assay using RNA-Seq that can provide accurate HLA genotyping and HLA allele-specific transcript expression in 7–8 h, a timeline short enough to perform the assay for deceased donors.

## Introduction

The application of next-generation sequencing (NGS) to human leukocyte antigen (HLA) typing has greatly reduced the costs and time required to achieve high-resolution HLA typing (Lind et al., 2010; Weimer et al., 2016; Cornaby et al., 2021). Currently, there are two major NGS platforms: short- and long-read sequencing. Short read platforms are characterized by sequencing reads between 50 and 300 bp, while long-read platforms can sequence reads from 150 to >2 Mb (Metzker, 2010; Jain et al., 2018). The application of long-read technology to HLA typing has been successfully demonstrated by several laboratories using DNA or RNA (Liu et al., 2018a; Liu, 20212018a; Lang et al., 2018; Montgomery et al., 2019; De Santis et al., 2020; Mosbruger et al., 2020). Long read technology is beneficial and applicable for HLA typing due to various advantages of the methodology, including shorter library preparation, real-time base calling, shorter sequencing times, and potential cost savings. The main focus for rapid HLA typing has been the development of an assay that yields high-resolution HLA typing in the time necessary for deceased donor allocation. Such an assay would enable improved virtual crossmatching and epitope analysis (Garcia-Sanchez et al., 2020).

A challenge with Oxford Nanopore Technologies (ONT) sequencers is the relatively higher error rate compared to traditional short-read sequencers. Short read platforms obtain reads with an accuracy approaching 99.99%; however, ONT sequencers achieve between 88–94% accuracy, depending on the chemistry and flow cell used (Metzker, 2010; Liu et al., 2021). One approach to reducing raw sequencing errors with ONT sequencing is the attachment of random oligomers of DNA to DNA or RNA before PCR amplification. The incorporation of unique molecular identifiers (UMIs) in the initial library preparation has become standard for single-cell analysis (Islam et al., 2014; Chen et al., 2018). Application of UMI to a library enables several benefits, including removal of PCR bias (Islam et al., 2014), quantitation of input molecules (Kivioja et al., 2011; Islam et al., 2014), and increased consensus read accuracy (Kou et al., 2016).

There is growing evidence for the importance of HLA expression in various diseases (Dendrou et al., 2018; Cornaby & Weimer, 2020; Cornaby et al., 2021) and allele-specific expression that influences T-cell activation (Gutierrez-Arcelus et al., 2020). Allele-specific HLA expression has been studied from single-cell sequencing data (Darby et al., 2020), and Johansson et al. (Johansson et al., 2021) using short-read sequencing, found variable allele-specific HLA mRNA expression. *HLA-DPB1* expression has been shown to influence graft versus host disease (Apps et al., 2013; Petersdorf et al., 2015). Recently, HLA expression has been shown to impact solid organ recipient and deceased donor immunologic compatibility (Badders et al., 2015; Montgomery et al., 2019). However, there are no current approaches to assess allele-specific HLA expression, protein, or transcript on deceased donors before transplantation. With allele-specific expression of donor antigens, virtual crossmatch predictions could be more accurate, and patient risk stratification could be assessed with more granularity.

Here, we describe an assay that provides high-resolution HLA typing and allele-specific HLA expression within the time constraints for deceased organ donor allocation. Using RNA and the application of UMI to quantify the mRNA HLA expression, the data demonstrate the variability of individual HLA allele groups.

## Materials and Methods

### Samples

Eighteen unique samples were collected in acid citrate dextrose (ACD) blood tubes from patients undergoing evaluation for solid organ transplantation and healthy donors. Patient and donor demographics are characterized in Table 1. The table describes the patient cohort age, sex, and race distribution. We had one patient among our cohort who did not identify with any race and is referred to as race unknown in Table 1.

**TABLE 1:** Cohort sociodemographic characteristics.

Characteristic	Values (N = 18)
Age, Median (Range)	53 (25–67)
Gender, Count (%)	
Female	11 (61.1)
Male	7 (39.9)
Race, Count (%)	
African American	11 (61.1)
European American	6 (33.3)
Unknown	1 (5.6)

### DNA Extraction and Quantitation

Genomic DNA (gDNA) was isolated from peripheral blood using Promega (Madison, WI) Maxwell RSC Whole blood DNA kits according to the manufacturer’s instructions. Briefly, 500 µL of the buffy coat or whole blood was added to the RSC extraction cartridge, and 75 µL of nuclease-free water (included in the kit) was added to the elution tube. The cartridge was loaded onto the Maxwell RSC, resulting in isolated gDNA that was ready for downstream applications. Genomic DNA did not undergo any additional purification and was quantitated using the Quantus Fluorometer and Quantiflour ONE dsDNA system by Promega (Madison, WI).

### RNA Extraction and Purification

Total RNA was isolated from peripheral blood using Promega (Madison, WI) Maxwell RSC SimplyRNA blood kits according to the manufacturer’s instructions. Briefly, 2.5 ml of whole blood was combined with 7.5 ml of cell lysis solution and then incubated for 10 min at room temperature. During this process, the red blood cells were lysed, and the white blood cells remained intact. The solution was spun for 10 min at 3,000 g, and the supernatant was discarded. Two hundred microliters of chilled 1-thioglycerol/homogenization solution, 200 µL of lysis buffer and 25 µL of Proteinase K were added to the cell pellet, mixed, and incubated at room temperature for 10 min. Ten microliters of DNase I solution was added to the RSC extraction cartridge in one of the chambers, and the cell lysate was added to another. Fifty microliters of nuclease-free water (included in the kit) was added to the elution tube. The resulting total RNA was used for downstream applications.

Some RNA samples underwent additional purification using the Promega (Madison, WI) ReliaPrep RNA Cleanup and Concentration System according to the manufacturer’s instructions. Membrane binding solution was added to the RNA at half the volume of the RNA (12.5 µL), and 100% isopropanol was added at one and a half times the volume of the RNA (37.5 µL). A series of washes and centrifugations were performed with the provided column wash solution and RNA wash solution, resulting in a final elution in 15 µL with nuclease-free water.

### Measurement of RNA Quality and Quantitation

RNA fragmentation and quantitation were determined using an Agilent (Santa Clara, CA) TapeStation 4200 RNA ScreenTape Assay following the manufacturer’s instructions. Briefly, 1 µL of RNA was added to 5 µL of sample buffer and loaded into the TapeStation. TapeStation plots the RNA fragment size (nt) against the sample intensity (FU) and generates an RNA integrity number (RIN). The TapeStation RNA ScreenTape Assay separates, images, and analyzes RNA from 50–6,000 nt. According to the manufacturer, the assay has a quantitative range of 25–500 ng/μL and a quantitative precision of 10% CV. The RIN functional range was 25–500 ng/μL. The RIN algorithm considers the peak height of the 18S peak to the background signal to calculate the RIN value for total RNA. RIN is on a scale of 1–10, where 10 represents highly intact RNA and a low RIN indicates a strongly degraded RNA sample.

### cDNA Synthesis

cDNA synthesis was performed using the SuperScript IV First-Strand Synthesis System (ThermoScientific, Waltham, MA). One hundred nanograms of total RNA was combined with 1 µL of 10 mM dNTPs and 1 µL of 50 µM Oligo d(T)_20_ primer and incubated for 5 min at 65°C and then placed on ice. A reaction tube was made consisting of 4 µL 5x SSIV buffer, 1 µL 10 mM DTT, 1 µL Ribonuclease Inhibitor and 1 µL SuperScript IV Reverse Transcriptase, resulting in reverse transcription (RT) Reaction Mix. The reverse transcription (RT) reaction mix was added to anneal RNA and then incubated at 53°C for 10 min followed by 80°C for 10 min. This resulted in cDNA being used in downstream applications.

### HLA Unique Molecular Identifier Assay

A series of PCRs and purifications were utilized to achieve the final enriched PCR product, which was an HLA-locus specific UMI amplicon. One microliter of cDNA was combined with 7.5 µL 2x Platinum SuperFi II Green PCR master mix (ThermoScientific), 0.6 µL each of HLA gene-specific forward and reverse UMI tagged primers at a concentration of 10 µM(Bettinotti et al., 2003; Lank et al., 2012), 0.5 µL 25 mM MgCl_2_ (ThermoScientific) and 4.9 µL of nuclease-free water (Ambion Inc., Austin, TX). Each sample had an amplification for *HLA-A, HLA-B, HLA-C, HLA-DRB1/3/4/5, HLA-DQA1, HLA-DQB1, HLA-DPA1,* and HLA*-DPB1*, generating 8 amplicons per sample. PCR was performed with an initial denaturation at 98°C for 3 min, followed by two cycles of denaturation at 98°C for 30 s, annealing with a touchdown from 66°C to 60°C with a ramp rate of 0.2°C/s holding at 60°C for 60 s, and extension at 72°C for 90 s, with a final extension of 72°C for 5 min. The first PCR was followed by an enzymatic removal of unincorporated gene-specific UMI primers by adding 1.5 µL of nuclease-free water and 0.75 µL each of thermolabile exonuclease I (New England Biolabs, Ipswich, MA) and Quick calf intestinal phosphatase (New England Biolabs) and then incubated at 37°C for 15 min followed by enzyme inactivation at 80°C for 2 min. To reduce off-target amplification, cleanup was performed using a 0.6x ratio of solid-phase reversible immobilization (SPRI) Select beads (Beckman Coulter, Brea, CA) and two washes with 80% EtOH. Each amplicon was eluted off the beads with 18 µL of nuclease-free water (Ambion Inc.). The eluate was used in the second round of PCR, where it was combined with 25 µL of 2x Platinum SuperFi II Green PCR master mix, 0.5 µL each of 10 µM UVP forward and reverse primers (Resource centre, 2017), 2 µL 25 mM MgCl_2_ and 4 µL of nuclease-free water. Thermal cycler conditions were initial denaturation at 98°C for 3 min, followed by five cycles of denaturation at 98°C for 30 s, annealing with a touchdown from 70°C to 63°C with a ramp rate of 0.2°C/s holding at 63°C for 10 s, extension at 72°C for 90 s, five cycles of denaturation at 98°C for 20 s with an extension of 72°C for 2 min and a final extension at 72°C for 5 min. Another round of SPRI bead cleanup using a 0.6x ratio and two washes with 80% EtOH was performed. The final round of PCR was performed using the eluate and 25 µL of 2x Platinum SuperFi II Green PCR master mix, 0.5 µL each of 10 µM UVP forward and reverse primers, which was the same master mix makeup that was used from the second round of amplification. The PCR conditions were initial denaturation at 98°C for 3 min, 25 cycles of denaturation at 98°C for 20 s with an extension at 72°C for 90 s, and a final extension at 72°C for 2 min. A final SPRI bead cleanup, as previously described, was performed using 80% EtOH washes.

### Quantification and Dilution of Amplicons

Each PCR product was analyzed with the QuBit 2.0 fluorometer (Thermo Fisher, Waltham, MA) dsDNA broad range (BR) assay. A portion of each PCR product was diluted with nuclease-free water to an estimated concentration of 20 ng/μL in 20 µL. A subsequent QuBit analysis was performed with the dsDNA BR assay to assure accurate concentrations in the library preparation.

### Library Preparation

HLA gene-specific amplicons for one sample (8 HLA loci) were combined into one tube in an equal molar ratio based on their femtomolar (fmol) concentration. A total of 350–400 fmol was targeted for each sample, and the volume was brought to 48 µL with nuclease-free water. Libraries were prepared according to the Native Barcoding Amplicons protocol using the Ligation Sequencing kit (SQK-LSK109) and Native Barcoding Expansion Kits (EXP-NBD104 and EXP-NBD114) from Oxford Nanopore Technologies (Oxford, United Kingdom) as follows. For a given sample, the HLA loci were pooled on an equimolar basis. In a PCR tube, 48 µL of pooled amplicon DNA, 3.5 µL NEB Next FFPE DNA Repair Buffer (New England Biolabs, Ipswich, MA), 2 µL NEB Next FFPE DNA Repair Mix (New England Biolabs), 3.5 µL Ultra II End-prep reaction buffer (New England Biolabs) and 3 µL Ultra II End-prep enzyme mix (New England Biolabs) were combined. Samples were mixed gently, spun down, and incubated at 20°C for 5 min and then 65°C for 5 min. A bead-based cleanup was performed using SPRI Select beads at a 1:1 ratio, and two washes were performed using 70% EtOH. Samples were eluted in 25 µL of nuclease-free water and then quantified using the QuBit dsDNA high sensitivity (HS) assay (ThermoScientific). Each sample was given a unique barcode utilizing the Native Barcoding Expansion Kit. A target of 150–300 fmol in a volume of 22.5 µL for each sample was used and combined with 2.5 µL of native barcode and 25 µL Blunt/TA Ligase Master Mix (New England Biolabs) and was mixed by pipetting and incubated at room temperature for 10 min. A bead-based cleanup was performed using SPRI Select beads at a 1:1 ratio (50 µL), and two washes were performed using 70% EtOH. Samples were eluted in 26 µL of nuclease-free water and quantified using the QuBit dsDNA HS assay. Equimolar amounts of each barcoded sample were pooled together into a 1.5 ml tube with a target of 350–400 fmol for the pool. The pool was brought up to 65 µL with nuclease-free water, to which 5 µL of Adapter Mix, 20 µL 5x NEBNext Quick Ligation Reaction Buffer (New England Biolabs), and 10 µL Quick T4 DNA Ligase (New England Biolabs) was added, mixed gently and incubated at room temperature for 10 min. Fifty microliters of SPRI Select beads was added to the tube, pipetted, and incubated on a rotator for 5 min. Tubes were placed on a magnetic rack for the beads to pellet, and the supernatant was pipetted off. Two washes using 250 µL each of short fragment buffer from the Ligation Sequencing Kit were performed. Final elution was performed using 15 µL of elution buffer from the Ligation Sequencing Kit and incubated for 10 min at room temperature. The final library was quantified with the QuBit dsDNA HS assay. Then, 100 fmol of the final library was used for sequencing. Sequencing was performed using a MinION R10.3 flow cell and loaded on the MinION Mk1C (Oxford Nanopore Technologies) with firmware version MinION FPGA 2.3.2 and MinKNOW version 21.02.2 using the high-accuracy basecalling module. Samples were run individually and in batches containing up to seven pooled samples.

### Reference HLA Genotyping

Isolated gDNA was HLA genotyped using the AlloSeq Tx17 assay (Care Dx, Brisbane, CA). Ten microliters of gDNA with a concentration of at least 10 ng/μL was utilized, and samples were prepared following the manufacturer’s instructions. First, a tagmentation step is performed on the gDNA, followed immediately by indexing PCR, which barcodes each sample. Double-tailed size selection and purification with SPRI beads (CareDx) were performed to target only the fragments of DNA of appropriate size. Samples were then combined (pooled) with up to 12 samples per pool. Pools were hybridized with the AlloSeq Tx17 probe panel with a slow ramp down from 98°C to 62°C ending with incubation at 62°C for at least 90 min and up to overnight. The 17 probes included in the AlloSeq Tx17 are designed to hybridize to the *HLA-A, -B, -C, -E, -F, G, -H, -DRB1, -DRB3, -DRB4, -DRB5, -DQA1, -DQB1, -DPA1, -DPB1, MICA,* and *MICB* loci. While the kit contains probes for these 17 loci, the 11 classical HLA loci were used for comparative analysis. A series of stringent washes with suppled buffers were performed post hybridization to capture the targets correctly. This was followed by a short enrichment PCR and postamplification cleanup per the manufacturer’s instructions using provided SPRI beads and 80% EtOH. Libraries were quantitated via Qubit dsDNA BR Assay. Sequencing was performed using the MiSeq platform (Illumina, San Diego, CA) with libraries denatured and diluted to 20 pM, and a 1% concentration of PhiX was added. A MiSeq cartridge with v2 chemistry and a microflow cell were utilized with 2 × 150 sequencing.

### Bioinformatics

All sequencing files for a patient were combined and used to generate consensus sequences based on the minimum number of reads grouped by UMI. Experiments used a minimum read of 1 per UMI (min1) or 10 (min10), which is a UMI group. Additionally, using a UMI group of 1 enables PCR deduplication. Adapters, primer sequences, and sample barcodes were removed using porechop (Wick et al., 2018; Hughes et al., 2020). UMI grouping was performed using vsearch (Rognes et al., 2016), and consensus sequences were generated using Medaka (version 1.1.3) (Xu et al., 2020). Consensus sequences generated were used for subsequent downstream analysis. The consensus sequence generation code is available at https://github.com/nanoporetech/pipeline-umi-amplicon/tree/cdna-no-mapping.

### RNA-Seq HLA Typing and Allele-Specific Transcript Quantitation

HLA typing was performed using (Liu et al., 2018b; Liu & Berry, 2020) a modified version of NGSEngine enabling cDNA as the input sequence (GenDx, Chicago, IL). When performing the assay, both pipelines were used to complement each other to achieve the genotyping results that were generated using NGSEngine without manual adjustments. The UMI count for each HLA allele was generated from the number of consensus reads, each associated with a unique UMI, which aligned to an allele using the Athlon2 pipeline (Liu, 2021). The alignment was implemented using minimap2 version 2.17 (Li, 2018) and cDNA sequences from the IPD-IMGT/HLA database v3.38. The accuracy of consensus reads from different UMI group sizes (i.e., min1 versus min10) was calculated as a percentage of the number of matching bases over the length of the alignment region.

### Normalization of Unique Molecular Identifier Counts and mRNA Transcripts

To normalize UMI counts across patients, as previously described in Johansson et al. (Johansson et al., 2021). Briefly, the sum of each HLA gene-specific UMI count was divided by the allele-specific UMI count, multiplied by the total UMI count for the patient, and scaled to 1 million reads. The output was then transformed using log base 2.

### Statistical Analysis

Statistical analysis was performed using R (version 4.1.1) and GraphPad Prism (version 9.3.1). One-way analysis of variance using Geisser-Greenhouse correction, followed by the Tukey multiple-comparisons test. Linear regression was used to evaluate correlation with R^2^ values derived from the Pearson correlation coefficient. The Kruskal–Wallis test, paired *t* test, independent *t* test, and Mann–Whitney U *t* test were utilized to determine statistical significance as appropriate. *p* values that were <0.05 were considered statistically significant.

## 4 Results

**FIGURE 1:**
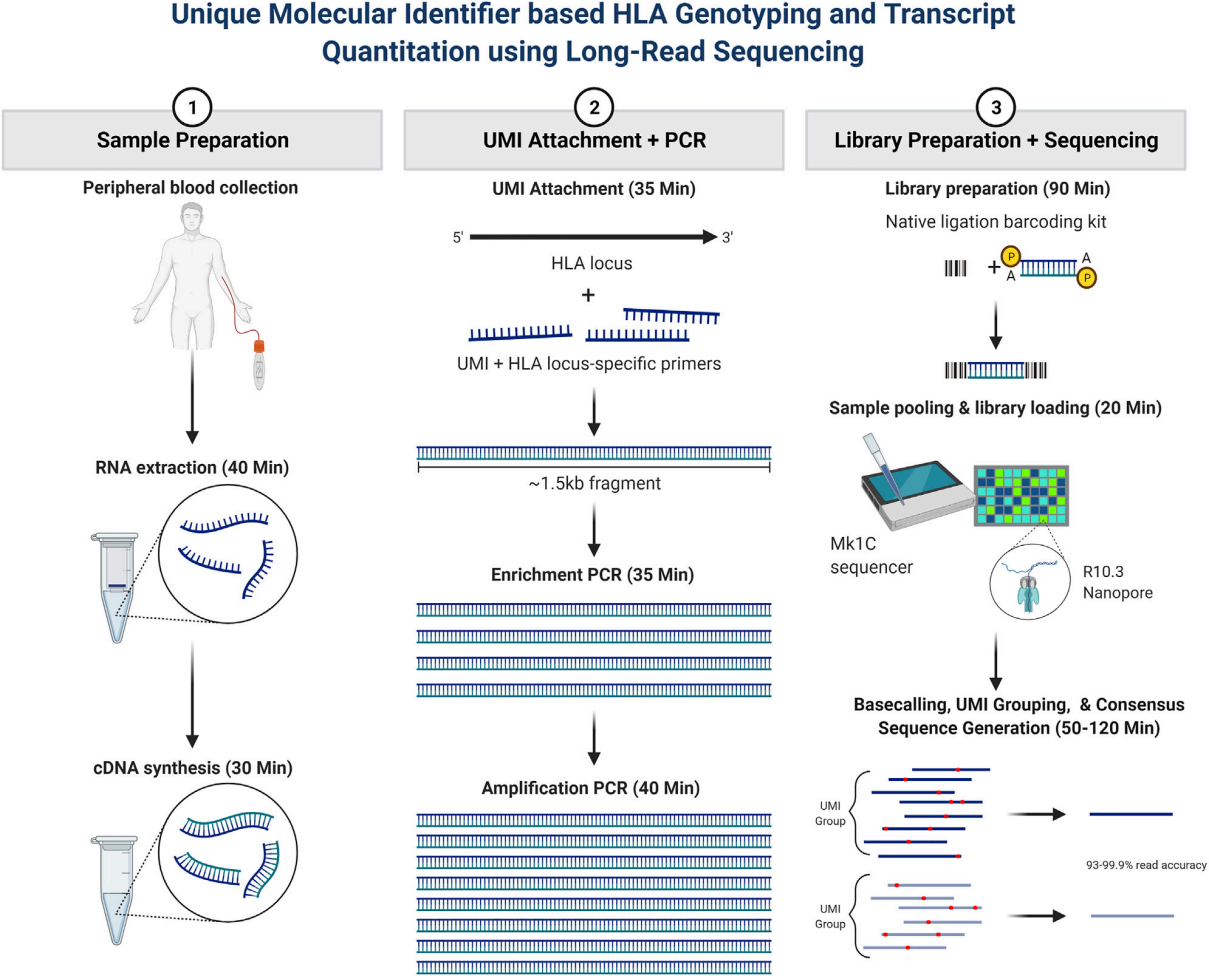
Patient sample workflow for RNA isolation, library preparation, and resultant HLA typing as described based on consensus sequence generation. In short, total RNA was isolated from peripheral blood, and a portion of the total RNA was purified. RNA fragmentation and quantitation were determined, and an RNA integrity number (RIN) was calculated for each sample. cDNA synthesis was then performed, after which PCR amplification and purification were utilized to achieve the final enriched HLA-locus specific UMI tagged amplicon. The HLA gene-specific amplicons for each patient were then used for library preparation and bar coding with the Oxford Nanopore Technologies (ONT) platform. Sequencing was performed using the MinION R10.3 Flow Cell and loaded on the MinION Mk1C using the high-accuracy base calling module. The NGSengine bioinformatics pipeline was then utilized to provide HLA typing and Athlon2 was used to generate HLA allele-specific UMI counts.

Our main study objective was to further evaluate whether long-read RNA sequencing could be utilized for rapid HLA genotyping and HLA RNA transcript expression. The overall assay schematic is shown in Figure 1. In short, total RNA was isolated from peripheral blood, and a portion of the total RNA was purified. RNA fragmentation and quantitation were determined, and an RNA integrity number (RIN) was calculated for each sample based on RNA fragment size and sample band intensity. The RIN value is on a scale of 1–10, where 10 represents highly intact RNA and a low RIN indicates a more fragmented RNA sample. cDNA synthesis was then performed, after which PCR amplification and purification were utilized to achieve the final enriched HLA-locus specific UMI tagged amplicon. The HLA gene-specific amplicons for each patient were then used for library preparation and barcoding for use on the Oxford Nanopore Technologies (ONT) platform. The final library with a target of 100 fmol was used for sequencing, which was performed using the MinION R10.3 Flow Cell and loaded on the MinION Mk1C using the high-accuracy base calling module.

In our cohort of 18 patients, the count of different alleles was as follows: 12 (*HLA-A*), 17 (*HLA-B*), 12 (*HLA-C*), 3 (*HLA-DPA1*), 12 (*HLA-DPB1*), 5 (*HLA-DQA1*), 5 (*HLA-DQB1*), 11 (*HLA-DRB1*), 3 (*HLA-DRB3*), 1 (*HLA-DRB4*), and 1 (*HLA-DRB5*). Our patient median age was 53 years old, with ages ranging from 25 to 67 years of age. Of our patients, 61.1% were female (Table 1). Our cohort included patients of African American and European American ethnicities, with the majority of our patients being of African American ethnicity or descent (61.1%).

### Metrics for cDNA-Based HLA Typing on the ONT Platform

**FIGURE 2:**
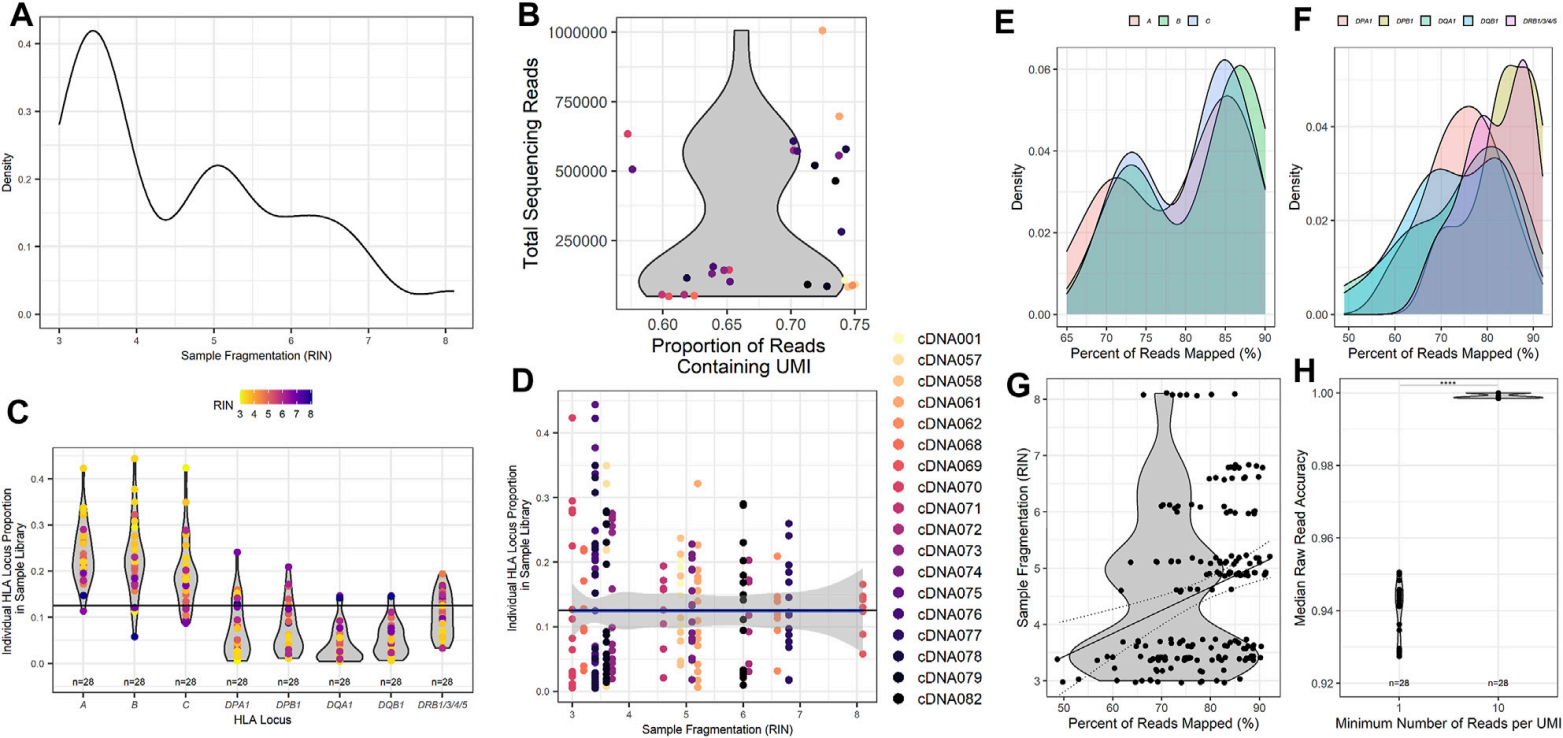
Metrics for HLA typing using unique molecular identifier-tagged RNA demonstrates that as fragmentation decreases, more reads are obtained for sequencing, and a greater proportion of reads can be mapped to HLA loci of interest. **(A)** Calculated RNA integrity numbers for each patient sample with their measured band density post isolation and clean-up. **(B)** Sample sequencing reads acquired and the proportion of the reads that contain recognized UMI barcodes. **(C)** The proportion of reads per classical HLA locus per patient sample as counted using UMI reads. **(D)** The proportion of reads for each HLA locus compared to the calculated RNA integrity number for each patient sample. **(E)** The percentage of cDNA reads obtained that could be mapped to the HLA loci obtained during sequencing and their respective calculated sample RNA integrity numbers. **(F)** Read accuracy calculated for the minimum number of UMIs required for each HLA locus and sample after concatenation of cDNA reads to consensus sequences. The percentage of **(G)** class I and **(H)** class II HLA-specific reads mapped compared to input isolated RNA measured band density (****p value <0.001).

To better assess the quality of RNA samples obtained from our patient samples, isolated RNA from each patient was measured by the TapeStation 4200 (Agilent), and an RNA Integrity Number (RIN) was calculated from the band density and sample fragmentation (Figure 2A). Our patient samples displayed a heterogeneous distribution of RIN values ranging from 3.0 to 8.1, with a median RIN value of 4.9. We also wanted to assess the efficiency of UMI tagging in the assay. To this end, the total reads for each patient were quantified, and the proportion of those reads that contained a UMI was determined (Figure 2B). Samples with higher total reads tended to have a higher proportion of UMI. More importantly, all patient sample sequences demonstrated that the majority of reads acquired were UMI labeled, with the proportion of reads that were UMI tagged ranging from 57.2% to 75.0% (Figure 2B).

To investigate and assess the collection of reads obtained from the classical HLA loci, we investigated the proportion of reads mapped to each of the classical HLA loci of the total reads sequenced (Figure 2C). Most samples exhibited the highest proportion of reads from the class I HLA loci *HLA-A, -B*, and *-C*, with class II HLA loci displaying a much lower proportion of total reads on average. To determine if sample RIN influenced the proportion of reads obtained across the classical HLA loci, the distribution of reads across the HLA loci was compared with the RIN value for each patient sample (Figure 2D). The data are heterogeneous. Nearly all samples, those with lower and higher RIN values, shared similar distributions of sequencing reads across the HLA loci, with a higher proportion of class I HLA loci compared to class II HLA loci.

To further evaluate whether relative amounts of RNA input might influence the alignment ability of the assay, we compared the TapeStation band density as measured by sample fluorescent intensity to the percentage of reads that were UMI identified and able to be mapped to HLA loci. All of the HLA loci demonstrated a similar trend with samples that had a higher band density also displaying a greater proportion of reads that were able to be aligned for class I (Figure 2E) and class II (Figure 2F). Since the band density demonstrated this trend, we wanted to ascertain if sample fragmentation influenced the mapping of reads to specific HLA loci. To do this, we investigated the percentage of reads that were aligned to HLA loci and compared those values to the patient sample RIN value (Figure 2G). Our samples showed a wide range of reads that could be mapped to HLA loci, ranging from 49% to 92%. While the R^2^ value derived from comparing the RIN value to the percentage of UMI tagged reads that are mapped to HLA loci is rather poor (Pearson r, R^2^ = 0.075), there does appear to be a trend that is noticeable when examining the results (Pearson r, *p* value = 0.0001). This comparison demonstrates that there could be a correlation between the fragmentation of samples and the ability of the samples to be successfully aligned to HLA loci. This trend implies that HLA loci from patient samples with a higher RIN result in a greater proportion of reads that can be successfully mapped.

### HLA Genotyping

As described in the Methods section and similar to other NGS sequencing assays, this assay utilizes UMIs to tag unique transcripts to quantity them and generate a consensus sequence of each unique transcript. The consensus sequences are generated by grouping sequences tagged with the same UMI. The number of reads within a specific UMI is used to set the minimum number of reads required to create the consensus sequence. To evaluate the accuracy of different UMI group thresholds, the accuracy of patient HLA typing using a minimum UMI read count of 1 (min1) and a minimum UMI read count of 10 (min10) were evaluated (Figure 2H). Not unexpectedly, the output consequence accuracy was significantly higher (*p* < 0.0001) for a min10 (98.7%) than for a min1 (95.9%) (Figure 2H).

To evaluate the typing concordance of our assay, we compared the resultant HLA genotype using the NGSengine bioinformatics pipeline to each patient’s reference typing results (Table 2). In our study, 18 unique patient samples, each of which was typed for 8 to 9 HLA loci per patient, provided a total typing of 314 HLA alleles. Overall, the accuracy was 99.68% (313/314), excluding the genotyping of a *DRB5* null allele. The only discrepant typing was one at *DQA1*, with the reference typing being a DQA1*05:05:01 and the assay resulting the allele as a DQA1*01:01:01.

**TABLE 2:** RNA-Seq HLA typing results utilizing NGSEngine

Sample ID	HLA-A		HLA-B		HLA-C		HLA-DPA1		HLA-DPB1		HLA-DQA1		HLA-DQB1		HLA-DRB1		HLA-DRB3/4/5	
	Reference	NGSEngine	Reference	NGSEngine	Reference	NGSEngine	Reference	NGSEngine	Reference	NGSEngine	Reference	NGSEngine	Reference	NGSEngine	Reference	NGSEngine	Reference	NGSEngine
cDNA001	02:01:01	02:01:01	35:01:01	35:01:01	03:04:01	03:04:01	01:03:01	01:03:01	02:01:02	02:01:02	01:01:01	01:01:01	05:01:01	05:01:01	01:01:01	01:01:01		
	11:01:01	11:01:01	40:01:02	40:01:02	04:01:01	04:01:01	-	-	03:01:01	03:01:01	01:04:01	01:04:01	05:03:01	05:03:01	14:54:01	14:54:01	3*02:02:01	3*02:02:01
cDNA057	02:01:01	02:01:01	07:06:01	07:06:01	15:05:02	15:05:02	01:03:01	01:03:01	04:01:01	04:01:01	02:01:01	02:01:01	02:02:01	02:02:01	11:02:01	11:02:01	3*02:02:01	3*02:02:01
	03:01:01	03:01:01	44:03:01	44:03:01	16:01:01	16:01:01	-	-	-	-	05:05:01	01:01:01	03:19:01	03:19:01	07:01:01	07:01:01	4*01:01:01	4*01:01:01
cDNA058	03:01:01	03:01:01	53:01:01	53:01:01	04:01:01	04:01:01	01:03:01	01:03:01	04:01:01	04:01:01	03:03:01	03:03:01	02:02:01	02:02:01	11:01:02	11:01:02	3*02:02:01	3*02:02:01
	-	-	57:02:01	57:02:01	18:02:01	18:02:01	03:01:01	03:01:01	105:01:01	105:01:01	05:05:01	05:05:01	03:19:01	03:19:01	-	-		
cDNA061	11:01:01	11:01:01	40:01:02	40:01:02	03:03:01	03:03:01	01:03:01	01:03:01	04:01P	04:01:01	03:01:01	03:01:01	03:01:01	03:01:01	11:01:01	11:01:01	3*02:02:01	3*02:02:01
	24:02:01	24:02:01	55:01:01	55:01:01	03:04:01	03:04:01	02:01:01	02:01:01	10:01P	10:01:01	05:05:01	05:05:01	03:02:01	03:02:01	04:04:01	04:04:01	4*01:03:01	4*01:03:01
cDNA062	30:01:01	30:01:01	35:01:01	35:01:01	04:01:01	04:01:01	01:03:01	01:03:01	04:01P	04:01:01	01:02:01	01:02:01	03:19:01	03:19:01	11:02:01	11:02:01	3*02:02:01	3*02:02:01
	30:02:01	30:02:01	42:01:01	42:01:01	17:01:01	17:01:01	-	-	03:01P	104:01:01	05:05:01	05:05:01	06:09:01	06:09:01	13:02:01	13:02:01	3*03:01:01	3*03:01:01
cDNA068	02:01:01	02:01:01	07:02:01	07:02:01	07:02:01	07:02:01	01:03:01	01:03:01	02:01:02	02:01:02	02:01:01	02:01:01	02:02:01	02:02:01	04:01:01	04:01:01	4*01:01:01	4*01:01:01
	03:01:01	03:01:01	44:03:01	44:03:01	16:01:01	16:01:01	02:01:01	02:01:01	11:01:01	11:01:01	03:01:01	03:01:01	03:02:01	03:02:01	07:01:01	07:01:01	4*01:03:01	4*01:03:01
cDNA069	30:01:01	30:01:01	42:01:01	42:01:01	06:02:01	06:02:01	02:01:01	02:01:01	01:01P	01:01:01	01:05:01	01:05:01	04:02:01	04:02:01	03:02:01	03:02:01	3*01:62:01	3*01:62:01
	68:01:01	68:01:01	58:02:01	58:02:01	17:01:01	17:01:01	02:02:02	02:02:02	11:01P	11:01:01	04:01:01	04:01:01	05:01:01	05:01:01	12:01:01	12:01:01	3*02:02:01	3*02:02:01
cDNA070	23:01:01	23:01:01	44:03:01	44:03:01	04:01:01	04:01:01	01:03:01	01:03:01	17:01:01	17:01:01	01:02:01	01:02:01	05:02:01	05:02:01	13:02:01	13:02:01	3*03:01:01	3*03:01:01
	26:01:01	26:01:01	-	-	-	-	02:01:01	02:01:01	18:01:01	18:01:01	-	-	06:09:01	06:09:01	13:31	13:31		
cDNA071	01:01:01	01:01:01	08:01:01	08:01:01	07:01:01	07:01:01	01:03:01	01:03:01	01:01:01	01:01:01	05:01:01	05:01:01	02:01:01	02:01:01	03:01:01	03:01:01	3*01:01:02	3*01:01:02
	30:02:01	30:02:01	47:03	47:03	07:18:01	07:18:01	02:02:02	02:02:02	06:01:01	06:01:01	-	-	-	-	-	-	3*02:02:01	3*02:02:01
cDNA072	03:01:01	03:01:01	07:02:01	07:02:01	04:01:01	04:01:01	02:02:02	02:02:02	01:01:01	01:01:01	01:02:01	01:02:01	03:19:01	03:19:01	15:03:01	15:03:01	5*01:01:01	5*01:01:01
	23:01:01	23:01:01	44:03:01	44:03:01	07:02:01	07:02:01	03:01:01	03:01:01	105:01:01	105:01:01	05:05:01	05:05:01	06:02:01	06:02:01	11:02:01	11:02:01	3*02:02:01	3*02:02:01
cDNA073	03:01:01	03:01:01	35:01:01	35:01:01	04:01:01	04:01:01	01:03:01	01:03:01	17:01:01	17:01:01	01:02:01	01:02:01	02:02:01	02:02:01	07:01:01	07:01:01	4*01:03:01	4*01:03:01
	30:01:01	30:01:01	44:03:01	44:03:01	-	-	02:01:01	02:01:01	18:01:01	18:01:01	02:01:01	02:01:01	06:02:01	06:02:01	15:03:01	15:03:01	5*01:01:01	5*01:01:01
cDNA074	23:01:01	23:01:01	44:03:01	44:03:01	04:01:01	04:01:01	01:03:01	01:03:01	02:01:02	02:01:02	01:02:01	01:02:01	02:02:01	02:02:01	11:01:02	11:01:02	3*02:02:01	3*02:02:01
	36:01	36:01	53:01:01	53:01:01	-	-	-	-	-	-	02:01:01	02:01:01	06:02:01	06:02:01	07:01:01	07:01:01	4*01:01:01	4*01:01:01
cDNA075	23:01:01	23:01:01	45:01:01	45:01:01	06:02:01	06:02:01	01:03:01	01:03:01	01:01:01	01:01:01	01:02:01	01:02:01	02:01:01	02:01:01	03:01	03:01:01	3*02:02:01	3*02:02:01
	23:17	23:17	-	-	16:01:01	16:01:01	02:01:08	02:01:08	02:01:19	02:01:19	05:01:01	05:01:01	05:02:01	05:02:01	11:01	11:01:02	3*03:01:01	3*03:01:01
cDNA076	68:02:01	68:02:01	15:03:01	15:03:01	02:10:01	02:10:01	02:01:01	02:01:01	05:01:01	05:01:01	01:01:02	01:01:02	02:02:01	02:02:01	07:01:01	07:01:01	4*01:01:01	4*01:01:01
	74:01:01	74:01:01	15:10:01	15:10:01	03:04:02	03:04:02	02:02:02	02:02:02	11:01:01	11:01:01	03:03:01	03:03:01	05:01:01	05:01:01	01:02:01	01:02:01		
cDNA077	02:01:01	02:01:01	49:01:01	49:01:01	07:01:02	07:01:02	01:03:01	01:03:01	04:02P	105:01:01	01:01:01	01:01:01	03:19:01	03:19:01	15:02P	15:02:01	5*01:08:01N	
	03:01:01	03:01:01	51:01:01	51:01:01	14:02:01	14:02:01	03:01:01	03:01:01	-	-	04:01:02	04:01:02	05:01:24	05:01:24	08:04:01	08:04:01		
cDNA078	02:01:01	02:01:01	42:01:01	42:01:01	05:01:01	05:01:01	01:03:01	01:03:01	04:02:01	04:02:01	01:02:01	01:02:01	03:01:01	03:01:01	04:01:01	04:01:01	4*01:03:01	4*01:03:01
	30:01:01	30:01:01	44:02:01	44:02:01	17:01:01	17:01:01	-	-	18:01:01	18:01:01	03:03:01	03:03:01	06:02:01	06:02:01	15:03:01	15:03:01		
cDNA079	01:01:01	01:01:01	37:01:01	37:01:01	03:04:01	03:04:01	01:03:01	01:03:01	01:01:01	01:01:01	01:02:01	01:02:01	05:01:01	05:01:01	10:01:01	10:01:01		
	02:01:01	02:01:01	40:01:02	40:01:02	06:02:01	06:02:01	02:01:02	02:01:02	04:01:01	04:01:01	01:05:01	01:05:01	06:02:01	06:02:01	15:01:01	15:01:01	5*01:01:01	5*01:01:01
cDNA082	11:01:01	11:01:01	15:10:01	15:10:01	01:02:01	01:02:01	01:03:01	01:03:01	04:01:01	04:01:01	01:01:01	01:01:01	05:01:01	05:01:01	01:01:01	01:01:01		
	33:03:01	33:03:01	56:01:01	56:01:01	03:02:02	03:02:02	-	-	18:01:01	18:01:01	01:02:01	01:02:01	06:02:01	06:02:01	15:03:01	15:03:01	5*01:01:01	5*01:01:01

### Expression of RNA Transcripts at Classical HLA Class I and Class II Loci

**FIGURE 3:**
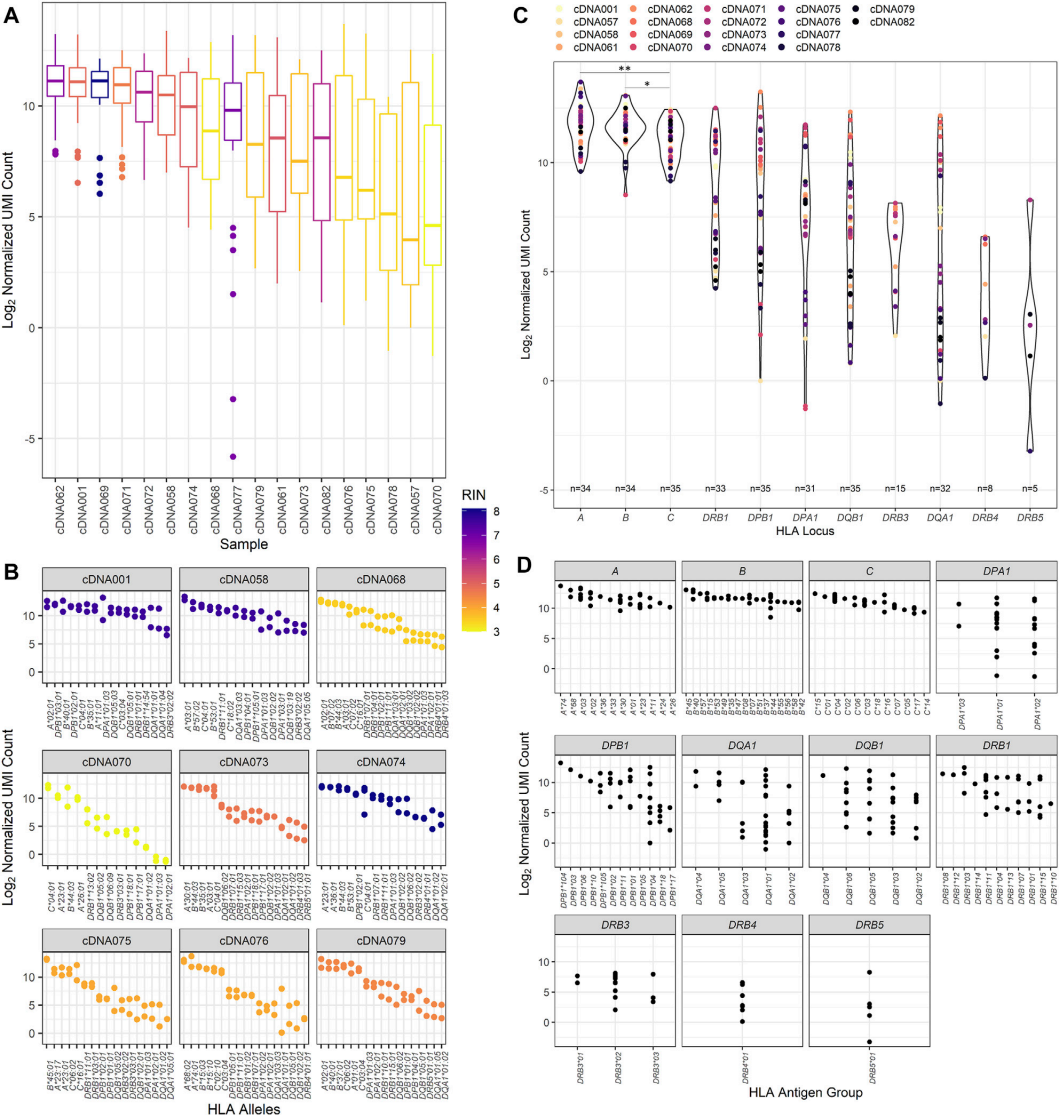
RNA expression of the patient cohort as counted by unique molecular identifier. **(A)** The normalized UMI count distribution for each patient sample corresponding to the calculated RNA integrity number. **(B)** The distribution of the normalized UMI counts across several representative patient HLA alleles and their corresponding calculated RNA integrity numbers. All nine of these patient samples were used to evaluate interassay precision, and these data are reflected on the respective patient plots. **(C)** Normalized UMI counts at each of the classical HLA loci (*p value <0.05, **p value <0.01). **(D)** The distribution of the UMI counts obtained for each HLA allele typed for the classical HLA loci for the study patient cohort.

Previous studies have evaluated using RNA-Seq to provide HLA typing. This study evaluated both the ability to provide HLA typing and HLA allele-specific expression using RNA-Seq. The expression of HLA transcripts was determined by counting the UMI-tagged HLA-specific reads and is described in detail in the methods section. This method was based on Johansson et al.(Johansson et al., 2021) and other allele-specific RNA-Seq expression studies. In this way, the UMI count for all patient samples was normalized for each HLA locus and read depth (Figure 3), the distribution of which was examined for each patient in our cohort (Figure 3A).

When comparing the expression of transcripts, higher transcript levels were associated with class I transcripts compared to class II transcripts (Figure 3B). This trend appears to be independent of sample RIN values, as samples with both high RIN and low RIN values exhibit this pattern of expression. Figure 3B also displays the interassay precision analysis. To this end, 16 of the 18 samples were repeatedly tested using the same input RNA as previously utilized for the primary patient runs. Identical typing results were obtained for the 16 patients, and no discrepancies were noted in the second field of HLA typing resolution. Evaluating the precision comparing the first and subsequent RNA-Seq HLA locus expression results, it was found that they were fairly similar for the class I loci, with the variation being only 7.37%, 6.48%, and 7.28% at the *HLA-A, -B*, and *-C* loci, respectively. There was more variation observed in the class II loci, with the lowest coefficient of variation of 24.27% noted for the *DRB1* locus. The expression of the other class II loci showed variation ranging from 34.32% at *DPB1* to 56.93% at the *DQA1* locus.

Of the class II loci, varying degrees of expression were observed. *DRB1* alleles demonstrated consistently high levels of transcripts and were significantly higher than *DQB1, DQA1*, and *DRB3/4/5* transcripts (Mann–Whitney U, *p* value = 0.0139, *p* value = 0.0054, and *p* value <0.0001, respectively) but did not have significantly different transcript levels compared to *DPA1* and *DPB1* alleles. Of interest, there was some heterogeneity in HLA expression observed among patients in our cohort for class I and class II (Figure 3C). For example, patient cDNA062 demonstrated higher expression of *DPB1, DQB1,* and *DQA1* transcripts compared to all other loci, including *HLA-A*, *-B,* and *DRB1* loci. A patient who exhibited expression patterns that were abnormal from the pattern of expression exhibited by the other patients was cDNA071. This patient had *DRB1, DPB1*, and *DPA1* transcript levels that were equally as high as the *HLA-A* and *-B* transcripts by UMI count.

We also wanted to examine the variability of expression between HLA alleles of the different loci (Figure 3D). It was noted that there was greater variance observed in class II loci for our cohort of patients compared to class I loci (Mann–Whitney U, *p* value = 0.0238), with class I loci demonstrating an average variation of 8.22% contrasted by the class II loci, which had an average expression variation of nearly 50.59%. Of the class II loci, the *DRB1* alleles demonstrated the least amount of variance, 30.12%, and *DRB3/4/5*, *DQB1*, and *DQA1* exhibited the greatest amount of variance at 59.40%, 51.51%, and 71.63%, respectively.

To investigate whether lower input amounts of cDNA might be responsible for the desperate variance observed between class I and class II, 9 of the 18 samples were repeated using twice the volume, doubling the amount of cDNA used initially. While the doubling of input cDNA did increase the UMI count of some of the class II transcripts, the increase was not significantly different for any of class I or class II HLA loci except for the *DPA1* locus (Supplementary Figure S1). Transcripts at the *DPA1* locus where transcript expression significantly increased by 18.96% on average (paired *t* test, *p* value = 0.034).

## Discussion

Our study presents an RNA-Seq assay approach that can provide HLA genotyping and mRNA expression levels of the classical HLA loci as calculated by UMI counting of the HLA loci reads, which has been demonstrated to be effective in other studies (Johansson et al., 2021). This method displays excellent accuracy and concordance with the NGS reference typing obtained using Illumina sequencing. The high accuracy is in part a result of setting a minimum threshold to ensure that a high enough number of unique UMIs are utilized to create the allele consensus sequence that is then used to derive the HLA genotyping. The assay had an overall HLA typing accuracy of 99.68% with one discrepant typing. Our discrepant typing was a DQA1*05:05:01 that was called a DQA1*01:01:01. Other samples with the same *DQA1* allele were typed using our RNA-Seq method. Upon investigation, we concluded that the discrepant typing results were likely related to sample collection, as repeat testing did not resolve this particular discrepancy. There was a second typing; however, it is not considered to be discrepant by this technique. This typing was a DRB5*01:08:01N where no transcripts were identified by RNA-Seq, even after repeat testing was performed. While mRNA transcripts of this null allele are likely produced, transcript levels were likely extremely low, possibly due to intracellular degradation as a result of its early transcript termination (Voorter et al., 1997; Barsakis et al., 2019). This is a limitation for any RNA-based HLA typing method compared to a genomic DNA NGS typing method. It is possible that null alleles with highly degraded or no mRNA expression would fail to be typed due to the nature of the typing methodology. This would have low to no clinical impact as null alleles do not demonstrate surface expression on donor tissue. As this is the case, not providing typing for null alleles would not impact clinical practice or patient outcomes in any way. However, the assay does perform well with relatively low transcript levels. Deceased donor typing or smaller batched runs should have less difficulty with low transcript levels as more reads can be acquired, increasing the depth of coverage, which should ameliorate this problem. We also had several samples where one allele portion was extremely low, ranging between 8.1% and 18.9% of all reads aligned to the locus. Eight of the nine (88.9%) samples where this low allele proportion was observed occurred at the *DQA1* locus with the remnant one at the *HLA-B* locus.

As we have observed in our patient cohort and from other studies investigating the expression of HLA loci, *DQA1* is one of the classical HLA loci with the lowest amount of mRNA expression (Johansson et al., 2021). As a result, it is not surprising that this HLA locus resulted in a discrepancy and that this was also the HLA locus that demonstrated the most allelic imbalance for several samples. Additionally, this could be the result of batching many patients per run using this method. If this method were performed for just one or two donors per ONT run, as would normally be performed for deceased donor typing by other methods, more reads could be obtained per patient sample, and the likelihood of dropout, allelic imbalance, or mistyping as a result of low transcript levels could be mitigated.

Aside from HLA genotyping, this RNA-Seq method also provides HLA allele mRNA transcript expression. Nearly all of our patient samples had a greater amount of UMI-counted reads from the class I HLA loci *HLA-A, -B*, and *-C*, with class II HLA loci displaying a lower proportion of total reads on average. These findings are consistent with what we would expect given the difference in the proportion of cells in the peripheral blood. We would expect that since our RNA was extracted from peripheral blood, there should be more class I mRNA transcripts than class II transcripts given the larger proportion of class I-expressing cells compared to class II-expressing cells. Our experimental results are similar to those of Johansson et al., who also utilized an RNA-Seq method to investigate HLA transcript levels (Johansson et al., 2021). As in their study, we found that class I locus transcripts were more abundantly expressed than class II transcripts. However, in our cohort of patients, we found that of our class I alleles, both the *HLA-A* and *-B* loci have higher expression than *HLA-C*. Their study cohort demonstrated lower transcripts of *HLA-A,* with *HLA-C* having the highest amount of transcripts of all the HLA loci that they evaluated. This difference is likely due to the population demographic differences between the studies. There is evidence that the HLA haplotype is more associated with *HLA-C* mRNA expression than with allotype or polymorphisms (Bettens et al., 2014). Additionally, it has been demonstrated that age likely influences HLA mRNA transcript levels for HLA class I loci (le Morvan et al., 2001).

This could also be related to the difference in methodology utilized between the studies. The method utilized by Johansson et al. uses a UMI that is attached to the 5′ end of the RNA transcripts using a template-switching oligo (TSO) during first-strand synthesis. This is then followed by amplification of the full-length cDNA. Our method performs cDNA synthesis as usual per the manufacturer’s instructions and then attaches the UMI to the 5′ end of the transcript after cDNA is synthesized and during PCR enrichment instead. We found that by attaching the UMI during the PCR enrichment step rather than during cDNA synthesis, we were able to obtain a higher UMI labeling efficiency. However, this difference in protocol might explain, at least in part, the different class I mRNA expression results observed in our patient samples.

However, the class II findings between this study and that of Johansson et al. are extremely similar. Our study provides mRNA expression data for the *DRB3/4/5* loci. While the expression of mRNA transcripts at the *DRB5* locus is fairly heterogeneous, the *DRB3* and *DRB4* loci demonstrate much less variance of expression. However, for most patients, these transcripts are some of the least abundant measured HLA loci examined. These results are concordant with studies that have investigated the expression of *DRB3/4/5* and compared it to the expression of mRNA transcripts at *DRB1*, mainly showing that *DRB1* transcripts are typically more abundant in lymphocytes than *DRB3/4/5* transcripts (Emery et al., 1993; Faner et al., 2010).

This assay does display increased variance between patient samples at the *DPB1, DPA1, DQB1, and DQA1* loci. This variance is likely a consequence of two factors. First, it is known that these loci exhibit lower expression than other HLA loci in certain patients and could also be due to allele-specific transcript expression, resulting in higher variance between patient samples. Second, these class II loci transcripts are only expressed by antigen presenting cells, which likely represent the minority of cells from which RNA was harvested. The proportion of these cells in the peripheral blood could also be different between patients, resulting in the high variance observed between patient samples that share the same DP and DQ alleles.

The mRNA expression obtained with this type of assay can contribute in several areas of transplant medicine. Quantification, or even just relative quantitation, of mRNA transcripts for each locus of a patient can assist in improving the evaluation for donor organ rejection based on the immunologic risk profile of the selected recipient. While elevated mRNA transcript levels do not always ensure elevated protein levels, there is an association that most elevated transcript levels result in elevated translation of transcripts into protein. As a result, understanding which transcripts are highly elevated can help predict which proteins would most likely be most prevalent on the surface of donor cells. As an example, in general, we expect that *HLA-C* is less expressed on the cell surface and is also typically less immunogenic; however, one of our patients displayed the highest count of mRNA transcripts for one of the *HLA-C* alleles compared to all other loci. These findings could suggest that the *HLA-C* loci for this patient might be more immunogenic due to the probably elevated amounts of allelic *HLA-C* expressed for the donor or that it might be more prudent to identify a matching donor at the *HLA-C* locus to alleviate potentially more risk at this HLA locus. This same principle could apply to any of the HLA loci where elevated levels of mRNA transcription might suggest an increased immunologic risk for donor-recipient pairs.

These assay results are extremely encouraging considering that both highly accurate HLA genotyping and RNA expression were able to be derived from samples that showed both low and high RIN values. Sometimes obtaining samples or utilizing different sample types for DNA- or RNA-based assays can be rather challenging. Understanding that this assay can provide reliable results even from highly fragmented RNA samples allows for the possibility that this assay could be used to provide HLA typing and gene expression on alternate sample types where RNA yield is very fragmented, such as in the case of RNA isolation from formalin-fixed paraffin-embedded (FFPE) tissue samples (Levin et al., 2020; Shi et al., 2021). This would allow laboratories to obtain HLA typing and transcript expression from tissue biopsy samples if there were the need to investigate patient physical crossmatch and HLA antibody test results. While our most fragmented samples with RIN values of 3 were able to achieve accurate HLA typing results, it is likely that samples with lower RIN values, exhibiting even more fragmentation, have an increased risk of HLA typing failure.

These results could assist in providing more accuracy and sensitivity for virtual crossmatch determinations (Garcia-Sanchez et al., 2020). By understanding the relative transcript levels or proportion of HLA allele transcript counts, centers would have the ability to use preexisting HLA antibody testing data to better identify which donor antigens are likely to result in a positive physical crossmatch given levels of mRNA expression (Montgomery et al., 2019; Weimer & Newhall, 2019). If a donor has relatively low amounts so mRNA transcripts of a given allele(s) that would otherwise have been assumed to be higher, a negative crossmatch determination could be made with higher confidence. The inverse of this would also be likely true as well, with abnormally increased mRNA transcripts observed, a positive crossmatch could be predicted with a higher level of confidence. This assay is a tool for enabling a better understanding of how allele-specific HLA transcript expression impacts transplant compatibility and outcomes. More studies are needed to elicit the exact nature of how HLA transcript levels impact immunologic compatibility.

A final advantage to utilizing this assay for clinical typing in the future is the advantage of turnaround times. Using the ONT platform, the assay has a turnaround time of 7–8 h from receipt of the sample in the laboratory to evaluation of final HLA typing results and HLA mRNA expression levels. This is shorter than the time required for current NGS methods used in the clinical transplant medicine laboratory, which normally requires approximately 18–24 h based on the NGS method utilized. Compared with AlloSeq hybrid capture NGS HLA typing, this assay is quite rapid (Figure 1), providing complete HLA typing and transcript expression in 6.5–7 h. Compared to the most rapid methods of obtaining deceased donor typing using real-time polymerase chain reaction (RT–PCR) or sequence-specific oligonucleotide (SSO), this assay is slightly more time consuming by several hours. However, the additional information gained, high resolution typing and donor HLA transcript expression would be beneficial and worth the additional assay time. With this RNA-Seq workflow timeline, a deceased donor could be typed, and HLA mRNA expression levels could be calculated to provide this additional information to better help transplant centers assess the immunologic risk of a donor-recipient pair. Additionally, donor transcript expression in conjunction with recipient anti-HLA antibody status could be utilized together to provide better virtual crossmatch predictions (Montgomery et al., 2019). The assay could also be utilized for typing living donors. Our patients were typed using batches of up to seven patients. However, the assay has the potential to be scaled up to accommodate a higher number of patient batches if necessary.

n this study, we describe a novel assay that provides high-resolution HLA typing and allele-specific HLA expression within the time constraints for deceased organ donor allocation. While the assay may not provide immediate benefits for the current organ allocation scheme, it would provide additional important information for transplant centers to utilize when deciding if an organ donor would be appropriate for their recipient. Currently, the United States United Network for Organ Sharing (UNOS) instructs that six antigen-matched organs be given elevated priority. While only a small portion of deceased donor kidneys are well matched, this assay would help provide additional information about the patient typing and potential protein expression that will allow individual centers to risk stratify their candidates for the organ offer with this additional information. The assay demonstrates reliable HLA typing performance even with samples that exhibit a high degree of fragmentation. This has major implications for the transplant laboratory, which often receives patient samples that are not of the highest quality but must be utilized due to time constraints or a lack of better patient samples. Additionally, using this method, it might be possible to provide typing for donors from FFPE slides or tissue samples that display a high degree of fragmentation. Utilizing RNA and the application of UMI counting to quantify the mRNA HLA expression, the data demonstrate the variability of individual HLA allele groups among individuals. The assay results may be essential in both solid organ and hematopoietic stem cell transplants as well other areas of medicine in providing additional information to further evaluate and risk-stratify donor and recipient pairs.

Our study is limited in that it used peripheral blood cells for HLA transcript expression. As described earlier in the article, this is likely why there is an increased amount of class I mRNA transcripts compared to class II transcripts. There is no way to adequately predict how the cellular components of each patient influenced the mRNA transcript levels calculated, but this likely influenced the transcript expression of each patient. A final limitation was that our study was restricted to only a small cohort of patients.

## Data Availability

The datasets presented in this article are not readily available because aspects of the data are under pending patent approval. Requests to access the datasets should be directed to eric_weimer@med.unc.edu.
