# Supplementary material for: Unique Molecular Identifier-Based High-Resolution HLA Typing and Transcript Quantitation Using Long-Read Sequencing
Source: Front Genet. 2022 Jun 13;13:901377. doi: 10.3389/fgene.2022.901377 (PMC9308011; doi:10.3389/fgene.2022.901377)
Supplement: Supplementary file 1 [file DataSheet1.docx]

Supplementary Material

## Supplementary Figures

**
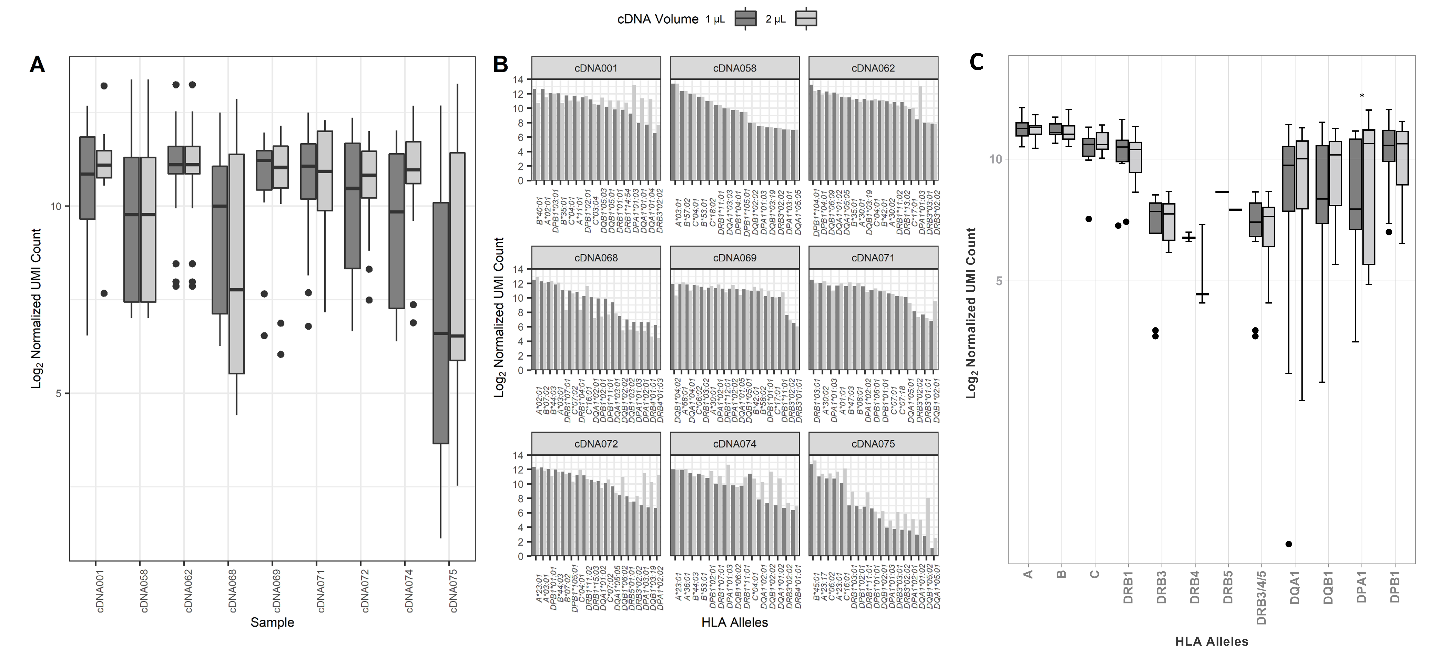
**

**Supplementary Figure 1.** **Sample and HLA loci mRNA transcript counts are extremely similar even after doubling the concentration of input cDNA.** Half of the cohort samples had the cDNA input doubled and the assay reperformed. The graphs illustrate the (A) normalized transcript levels observed for each patient sample, (B) each HLA allele, and (C) each of the classical HLA loci investigated (*P-value < 0.05).


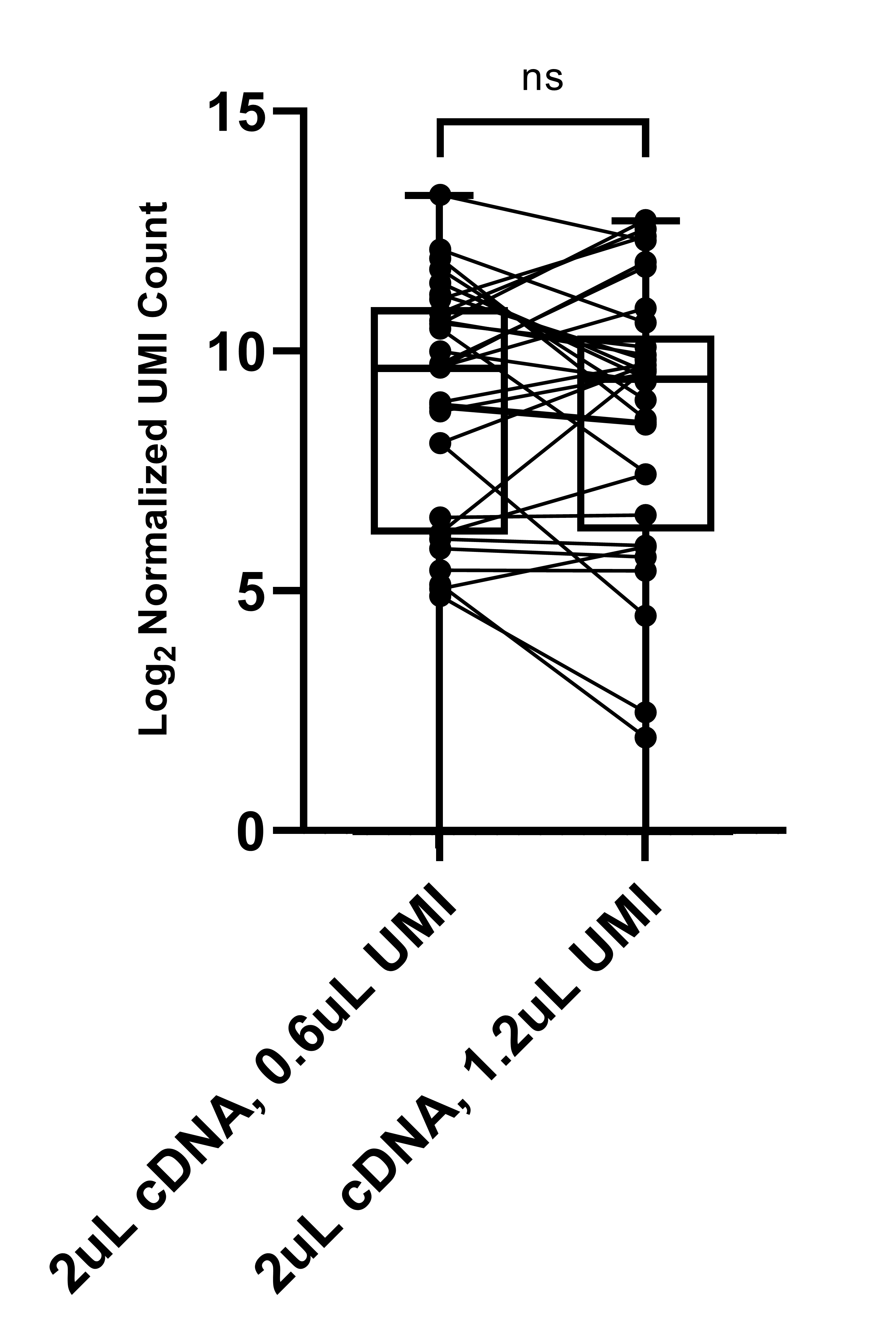
**Supplemental Figure 2. Impact of varying UMI primer on transcript expression.** Two of the samples were tested using the RNA-Seq HLA typing assay described utilizing varying amounts of assay UMI primers for each HLA locus (x-axis). These results compare the expression (y-axis) calculated for these samples for each HLA allele. Paired analysis was performed using the Wilcoxon paired test (ns = Not Statistically Significant).
